# A Universal Photochemical Method to Prepare Carbohydrate Sensors Based on Perfluorophenylazide Modified Polydopamine for Study of Carbohydrate-Lectin Interactions by QCM Biosensor

**DOI:** 10.3390/polym11061023

**Published:** 2019-06-10

**Authors:** Lili Guo, Shuang Chao, Pei Huang, Xiukai Lv, Quanquan Song, Chunli Wu, Yuxin Pei, Zhichao Pei

**Affiliations:** 1 Shannxi Key Laboratory of Natural Products & Chemical Biology, College of Chemistry & Pharmacy, Northwest A & F University, Yangling 712100, China; guoll513@163.com (L.G.); chaoshuang@nwafu.edu.cn (S.C.); huangpei723@163.com (P.H.); Lvxiukai@nwafu.edu.cn (X.L.); quanquans1995@163.com (Q.S.); peiyx@nwafu.edu.cn (Y.P.); 2 School of Pharmaceutical Sciences, Zhengzhou University, 100 Kexue Avenue, Zhengzhou 450001, China

**Keywords:** perfluorophenylazide, polydopamine, underivatized carbohydrates, QCM biosensor, carbohydrate-lectin interactions

## Abstract

A universal photochemical method to prepare carbohydrate sensors based on perfluorophenylazide (PFPA) modified polydopamine (PDA) for the study of carbohydrate–lectin interactions by a quartz crystal microbalance (QCM) biosensor was developed. The PFPA was immobilized on PDA-coated gold sensors via Schiff base reactions. Upon light irradiation, the underivatized carbohydrates were inserted into the sensor surface, including mannose, galactose, fucose and N-acetylglucosamine (GlcNAc). Carbohydrate sensors were evaluated for the binding to a series of plant lectins. A kinetic study of the interactions between mannose and concanavalin A (Con A), fucose and Ulex europaeus agglutinin I (UEA-I) were performed. This method can eliminate the tedious modification of carbohydrates, improve the experimental efficiency, and reduce the experimental cost, which is of great significance for the development of QCM biosensors and the study of biomolecular interactions.

## 1. Introduction

Carbohydrates play important roles in cell communication, apoptosis, immune response and infection by interacting with proteins and other biomolecules [1,2,3]. The quartz crystal microbalance (QCM) is an ultrasensitive weighing device, consisting of a thin disk of single crystal quartz, with metal electrodes deposited on each side of the disk. The crystal can be made to oscillate at its resonant frequency, *f*, when connected to an external driving oscillator circuit [4]. Biosensors based on QCM have proven to be an efficient and powerful instrument for label-free detection of biomolecular interactions in real-time, permitting the determination of the kinetics and affinity parameters of the interaction, including the dissociation rate constant, association rate constant, and affinity constant. It can be used for analysis of interactions between protein and carbohydrate with carbohydrate sensors, protein sensors, and cell sensors [5,6,7,8,9,10,11,12]. Therefore, as an efficient analysis tool, QCM sensor technology has attracted widespread attention, especially carbohydrate sensors for carbohydrate-protein interactions [13,14]. Lectins are a heterogeneous group of proteins, have at least one non-catalytic domain that recognize selectively and bind reversibly to specific free carbohydrates or glycans presenting on glycoproteins and glycolipids. The interactions between carbohydrate and lectin are specific and the structure of the carbohydrates will not be altered [15]. QCM biosensor can be used to monitor the binding affinities of carbohydrate and lectin. The binding between an immobilized lectin Con A and polysaccharides was researched using a QCM sensor [16]. Moreover, we successfully immobilized amino-carbohydrates on the surface of the sensor to detect the interactions of carbohydrate-lectin [12].

The efficient immobilization of carbohydrates on a sensor surface is a primary condition for the preparation of QCM carbohydrate sensor surface. There are usually two strategies to immobilize the carbohydrate on the sensor surface, which include covalent immobilization and non-covalent immobilization [17]. The application of non-covalent immobilization is limited because of its poor stability. However, the covalent immobilization method connects the carbohydrate to the basal surface by a covalent bond, which leads to a high stability and wide application. In fact, the preparation of carbohydrate sensors by covalent immobilization frequently requires multi-step synthesis of the carbohydrates, which is generally not applicable to the direct immobilization of underivatized carbohydrates on the sensor surface [15], where the process for multi-step modification of the carbohydrates is usually time consuming [18,19,20,21]. Therefore, it is thus highly desirable to develop a universal method which is applicable to the preparation of carbohydrate sensors by directly using the underivatized carbohydrates without any modification.

Perfluorophenylazide (PFPA) has special chemical properties. The azide functionality of PFPA can be converted to nitrene under irradiation or heating that can insert C-H, N-H or C=C bond to form a new covalent bond [22]. It has thus been used to thermo- and photochemically lead into functional groups in a range of substances, such as proteins, carbohydrates, and nanoparticles, since it can significantly improve insertion efficiency [23,24,25,26,27]. The versatility of PFPA makes it to be an ideal choice for surface modification. Tyagi et al. [3] used thiol-modified PFPA to covalent immobilize underivatized carbohydrates to gold surfaces by a fast photochemical reaction to prepare Au surface plasmon resonance (SPR) sensors, where the arrays for protein-binding effects were described by SPR imaging.

Polydopamine (PDA) is a well-known biomimetic adhesive material, which can be covered on the surfaces of any kind of materials in a simple way [28,29]. Dopamine (DA) is able to react with oxygen to self-polymerize in an alkaline solution and form a thin PDA layer, which can steadfastly attach to a wide range of substrates, such as rocks, metals, polymers and wood. Under alkaline conditions [30,31], the catechol group of PDA will be oxidized to the ortho-benzoquinones, which can react with the nucleophilic group by Michael addition reactions and Schiff-base reactions, such as amino and thiol groups [32]. Compared with traditional methods [33,34], this method has the advantages of a mild reactive condition and user-friendly control, which is widely used on various material surfaces. In our previous work, we successfully prepared a protein sensor which was functionalized by PDA to analyze the interactions between proteins [35]. Moreover, we successfully immobilized amino-modified carbohydrates on the surface of PDA-coated sensors to detect the interactions of carbohydrate-lectin. However, the process for multi-step modification of the carbohydrates was usually time-consuming [12].

In this study, carbohydrate sensors were prepared with underivatized carbohydrates based on PFPA modified PDA sensors by a photochemical fabrication, which avoid the multi-step modification of the carbohydrates. The prepared carbohydrate sensors were further used to evaluate the interactions of carbohydrate-lectin by QCM biosensor. This method can eliminate the tedious modification of carbohydrates, improve the experimental efficiency, and reduce the experimental cost, which has great significance for the development of QCM and the study of biomolecular interactions. Firstly, PFPA derivatives were immobilized on the PDA-coated gold sensor surface by Schiff-base reactions, and subsequently four different underivatized carbohydrates (mannose, galactose, fucose and GlcNAc) were inserted on the sensor surfaces by photochemical fabrication under light conditions. Secondly, the prepared carbohydrate sensors were evaluated for the binding to a series of plant lectins, which include wheat germ agglutinin (WGA), soybean agglutinin (SBA), peanut agglutinin (PNA), UEA-I and Con A. In addition, two kinds of carbohydrates and their corresponding lectins were selected for kinetic study. This research provides a universal method for the preparation of carbohydrate sensors based on PFPA modified PDA sensors by photochemical covalent immobilization of underivatized carbohydrates, which can be used for the analysis of carbohydrate-lectin interactions by QCM biosensors.

## 2. Materials and Methods

### 2.1. Materials

2,2′-(Ethylenedioxy)bis(ethylamine), dopamine hydrochloride and D-mannose were obtained from Xianding Biotechnology Co. (Shanghai, China). Methyl pentafluorobenzoate and aminoacetic acid was obtained from Shanghai Aladdin Bio-Chem Technology Co. (Shanghai, China). *N*-acetyl-d-glucosamine and l-fucose were obtained from Shanghai Macklin Biochemical Co. (Shanghai, China). Bovine serum albumin (BSA) and d-galactose were obtained from Energy Chemical (Shanghai, China). SBA, WGA, PNA, and UEA-I were obtained from Vector Laboratories (Burlingame, CA, USA). Con A, concanavalin A fluorescein conjugate (FITC-Con A) and tris(hydroxymethyl)amino-methane (Tris) were obtained from Sigma-Aldrich (Shanghai, China).

**Figure 1:**
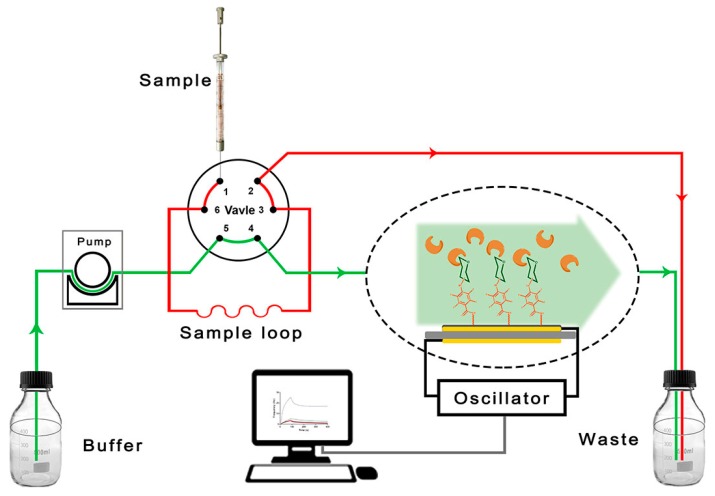
Scheme of the Attana quartz crystal microbalance (QCM) biosensor flow-through system.

^13^C, ^19^F and ^1^H NMR spectra were recorded by using a Bruker AVANCE III HD 500 MHz Spectrometer (Karlsruhe, Germany) in CDCl_3_. SEM-EDX were obtained using the S-4800 instrument (Hitachi Ltd.) with an accelerating voltage of 10.0 kV. The photochemistry in this work was undertaken with a Rayonet PRP-100 photochemical reactor (Branford, CT, USA). The experiments were carried out using an Attana QCM Cell-A200 (Stockholm, Sweden) (Figure 1). An Attana gold sensor (Stockholm, Sweden) was used for further modifications to obtain a carbohydrate sensor.

### 2.2. Sensors Cleaning

QCM gold sensors were placed in a 50 mL beaker and immersed with 1% NaClO solution. The beaker was shaken slowly to clean the sensors for 2–3 min, and then the sensors were cleaned with 1% NaClO solution 2–3 times until the surface of the sensors were bright yellow. Furthermore, the sensors were washed with Milli-Q water 4–5 times and dried with nitrogen stream.

The sensors mentioned above were cleaned for 1 h in 30% H_2_O_2_ (0.4 mL) and H_2_SO_4_ (1.2 mL) at 50 °C. At the same time, the beaker was shaken continuously during this process. The sensors were washed with a large amount of Milli-Q water until neutral, then washed with ethanol three times and dried with nitrogen stream.

### 2.3. Modification of Polydopamine on the Sensor Surfaces

50 μL dopamine solution (2 mg/mL) in 10 mM Tris buffer (pH 8.5) was added to the gold surfaces at room temperature for 1.5 h. After incubation, the sensor surfaces were washed with Milli-Q water and dried with nitrogen stream to obtain the polydopamine-modified QCM sensors.

### 2.4. Synthesis of Amino-PFPA

Compound **7** was synthesized via the previously reported method, which was shown in the Supporting Information.

### 2.5. Preparation of the PFPA-modified Sensors

Compound **7** (5 mg) was dissolved in 100 μL PBS of pH 8.5. The solution (50 μL) was added to the surfaces of the modified PDA-coated sensors, and incubated in a wet box for 4 h, then the sensor surfaces were further rinsed with PBS in order to remove compound **7,** which was unimmobilized on the surfaces.

### 2.6. Preparation of Carbohydrate Sensors

50 μL carbohydrate solution (100 mM) was added to the PFPA-modified sensors, after which the photochemical fabrication of PFPA was carried out under ultraviolet light of 300 nm for 10 min. The sensor surfaces were washed three times with PBS to remove uninserted carbohydrates and dried with nitrogen stream to obtain the carbohydrate sensors.

### 2.7. SEM-EDX Measurement

The samples were characterized by SEM-EDX to analyze the surface chemical composition of sensor surfaces after drying overnight.

### 2.8. QCM Analysis of Carbohydrate-Lectin Interactions

The sensors were docked into the QCM Cell-A200 and equilibrated under a continuous flow rate of running buffer (100 μL/min). Subsequently, the flow rate was reduced to 25 μL/min to study interactions between carbohydrate and lectin. To block the areas which were not react with carbohydrate, BSA (100 μg/mL) was subsequently injected over the carbohydrate sensor surfaces until the surfaces were saturated. Then, 100 µg/mL lectins were injected into the surface of the sensors. The resonant frequency of the quartz crystal could be recorded with the Attester software in real time, just as the frequency shift (Δ*f*) coupled to the association or dissociation. The interactions of carbohydrate and lectin were monitored for an association period of 84 s and a dissociation period of 300 s. The evaluation software of the Attana QCM Cell-A200 was used to record data. Finally, the bound lectin was removed by injecting 10 mM glycine of pH 1.5 to regenerate the sensors. Attester Evaluation software was used to analyze the data.

## 3. Results and Discussion

**Figure 2:**
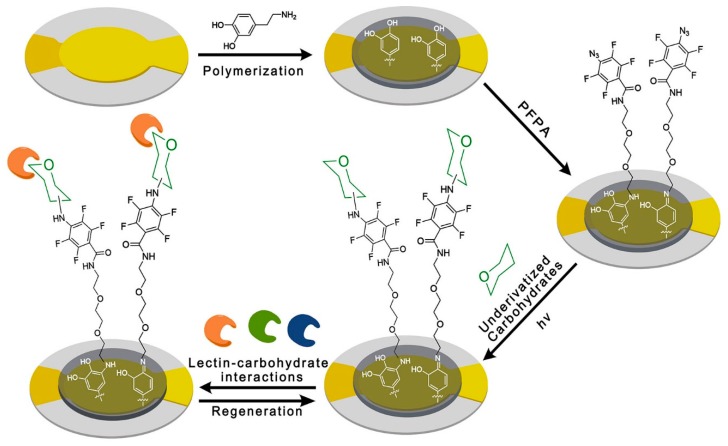
Schematic illustration of preparing the carbohydrate sensor, based on the universal method.

The QCM gold sensor surfaces were prepared employing a three-step process. As shown in Figure 2, firstly, in alkaline environment, the gold surfaces were fabricated with PDA via dopamine self-polymerization. After coating of the PDA, the PFPA with an amino group was immobilized on the surfaces of the PDA-coated sensor by Schiff-base reactions. Finally, PFPA of photochemical fabrication was used to immobilize underivatized carbohydrates on the sensor surfaces under an ultraviolet light of 300 nm. In this work, the carbohydrate-lectin interactions were monitored by QCM biosensor. Setting the rate of flow to 25 μL/min of running buffer (10 mM PBS of pH 7.4), the interaction between carbohydrates and different lectins was detected. In addition, the kinetic properties of the interactions between carbohydrate and lectin were further performed, where we selected two kinds of carbohydrates for the kinetic study. The interactions between carbohydrate and lectin can be monitored by QCM biosensor, permitting the determination of the affinity and the kinetics parameters of the interactions.

### 3.1. SEM-EDX Analysis of the Sensor Surfaces

**Figure 3:**
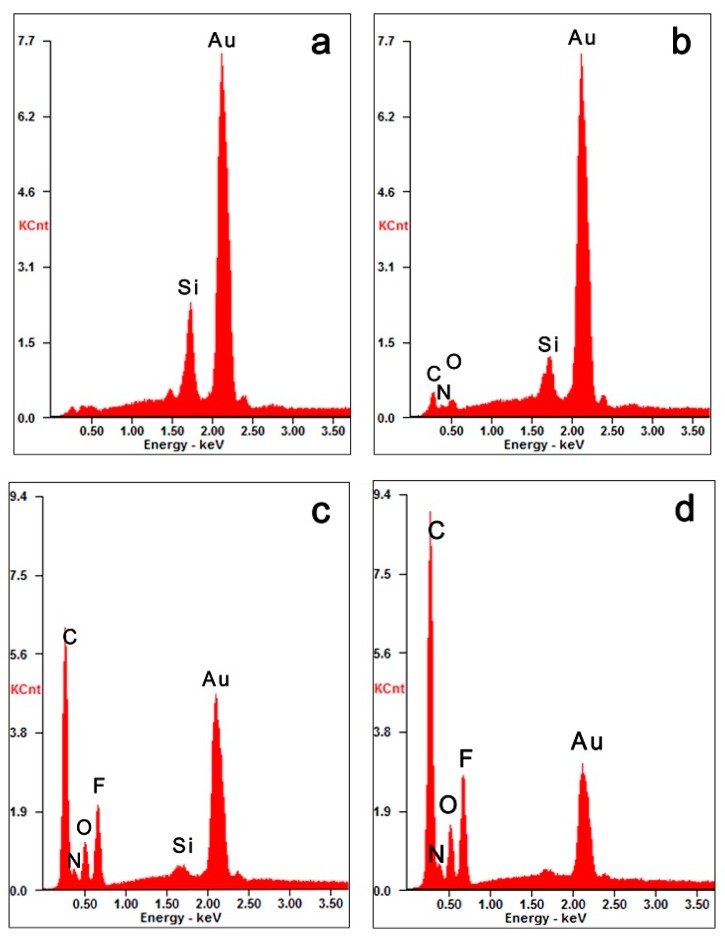
SEM-EDX spectra of different sensor surfaces: (**a**) unmodified gold sensor surface; (**b**) polydopamine (PDA) coated sensor surface; (**c**) perflourophenylazide (PFPA) immobilized sensor surface; (**d**) carbohydrate sensors.

As shown in Figure 3, the chemical composition of these sensor surfaces can be analyzed by SEM-EDX [36]. Based upon the spectra analysis of SEM-EDX, the elemental peak of C, N and O appeared on the PDA-coated gold sensors, compared with the element composition of gold sensors (Figure 3a). The spectrum of SEM-EDX indicated the evident signal of the PDA’s atomic composition (Figure 3b). As shown in Figure S6 of Supporting Information, the region of peaks below 1 keV for Figure 3a,b were enlarged. Compared with the sensor surfaces coated with PDA, the SEM-EDX spectral signal of the chemical composition F appeared on the sensor surfaces after immobilizing compound **7** (Figure 3c), which indicated that the PFPA was connected to the surfaces of the sensor successfully. As shown in Figure 3d, the spectral signal of the chemical composition C and O improved markedly compared to the PFPA-modified sensor, which indicated the immobilization of the carbohydrate on the sensor surfaces.

### 3.2. FITC-Con A Immobilization on the Carbohydrate Sensor Surface and Regeneration Result

**Figure 4:**
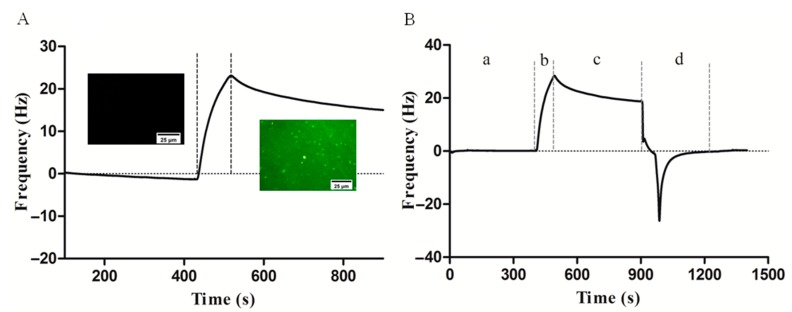
Binding of FITC-Con A to carbohydrate sensor (**A**) and QCM analysis of the binding of lectin with carbohydrate immobilized on the PFPA-modified sensor surface (**B**). Frequency shift was recorded during the association (84 s) and dissociation (300 s) phases of the interaction between lectin and carbohydrate, as well as the subsequent desorption of the remaining bound lectin by an injection of the 10 mM glycine pH 1.5. (**a**) baseline; (**b**) association; (**c**) dissociation; (**d**) regeneration.

To verify the interactions between carbohydrate and lectin, FITC-Con A with green fluorescence was used to observe whether the carbohydrate on the sensor surface binding with lectin successfully. When the baseline was stable (frequency shift less than 0.2 Hz/min), BSA was injected into the carbohydrate sensor surface to block the area where PFPA did not react with carbohydrate until the sensor surface was saturated. Then, 100 µg/mL FITC-Con A was injected and observed by a fluorescence microscope to prove interactions between carbohydrate and lectin. As shown in Figure 4A, the binding response of interactions between mannose and FITC-Con A was recorded, resulting in a frequency shift about 20 Hz. Before and after the injection of the FITC-ConA, a fluorescence microscopic evaluation was performed. Before injection, only a black field was observed, while a green fluorescence could be observed after injection of FITC-ConA onto the carbohydrate sensor surface. Furthermore, regeneration of the carbohydrate sensor surface was carried out, where the bounded Con A can be removed by injecting 10 mM glycine of pH 1.5 (Figure 4B). As can be seen, the baseline reverted to its original state, which indicates that the carbohydrate sensor surface was regenerated successfully.

### 3.3. QCM Measurements of Carbohyhdrate-Lectin Interactions

According to the reports, Con A, SBA, PNA, WGA, and UEA-I can specifically bind mannose, galactose/GalNAc, galactose/glucose, GlcNAc, and fucose, respectively [37,38,39,40,41,42]. In this study, we chose four carbohydrates (mannose, galactose, GlcNAc and fucose) to study five lectins interactions with carbohydrates, respectively. 

**Figure 5:**
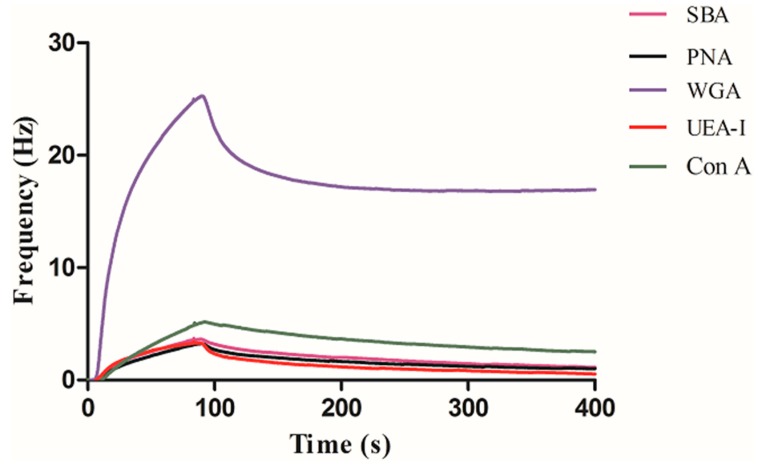
Interaction of GlcNAc with a series of lectins.

The prepared carbohydrate sensors were inserted in the Attana QCM Cell-A200 biosensor. When stable baseline was achieved at 25 μL/min of the PBS running buffer, we blocked the areas which did not react with carbohydrate by BSA. The BSA was repeatedly injected to the carbohydrate sensor surfaces until the surfaces were saturated. Then a series of lectins of 100 μg/mL WGA, SBA, PNA, UEA-I, and Con A were injected to detect the interactions of carbohydrate-lectin, respectively. After each injection of the lectin, 10 mM glycine (pH 1.5) was injected to the surface for regeneration, and the binding lectin was removed after the cycle. We used Attester software to realize real-time monitoring of carbohydrate with lectin interactions and regeneration processes. Figure 5 shows frequency change of five different lectins (WGA, SBA, PNA, UEA-I, Con A) binding to GlcNAc-modified sensor chip surface. As can be seen, the binding of WGA is the highest, at about 20 Hz, indicating that other lectins are basically not bound except for non-specific adsorption.

**Figure 6:**
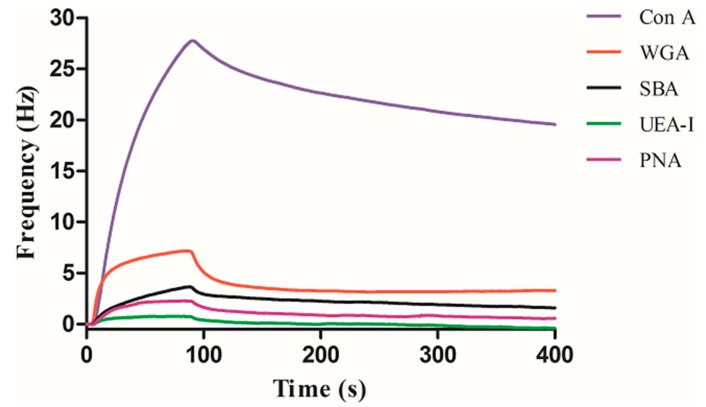
Interaction of mannose with a series of lectins.

**Figure 7:**
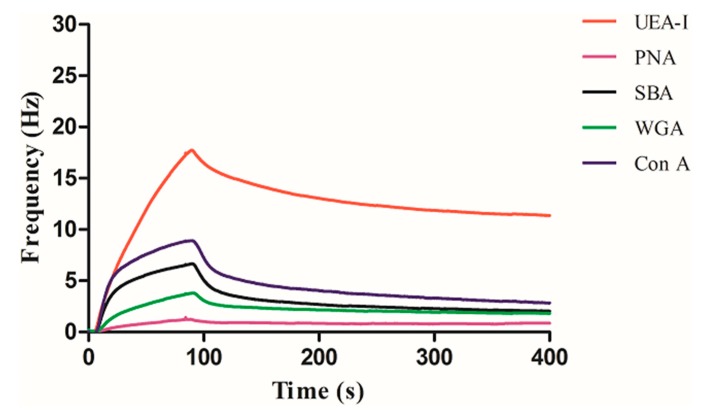
Interaction of fucose with a series of lectins.

**Figure 8:**
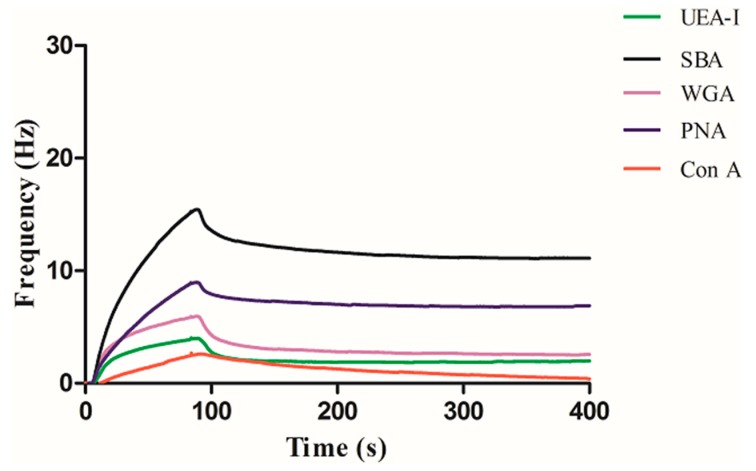
Interaction of galactose with a series of lectins.

The binding results between the other three carbohydrates and five different lectins were represented in the Figure 6, Figure 7 and Figure 8, respectively. As can be seen in Figure 6, Con A has the highest binding with mannose, while the other lectins were basically not bound except that a small amount of non-specific adsorption was detected. As can be seen in Figure 7, UEA-I has the highest binding with fucose, while the other lectins were basically not bound except that a small amount of non-specific adsorption was detected. As can be seen in Figure 8, SBA has the highest binding with galactose, followed by PNA. This is because both SBA and PNA can bind to galactose, but the binding strengths are different, while the other lectins are not bound except for a small amount of non-specific adsorption. The experimental results show that the interactions between carbohydrate and lectin are specific, which are consistent with the results obtained by other methods, indicating the universality of this method.

**Figure 9:**
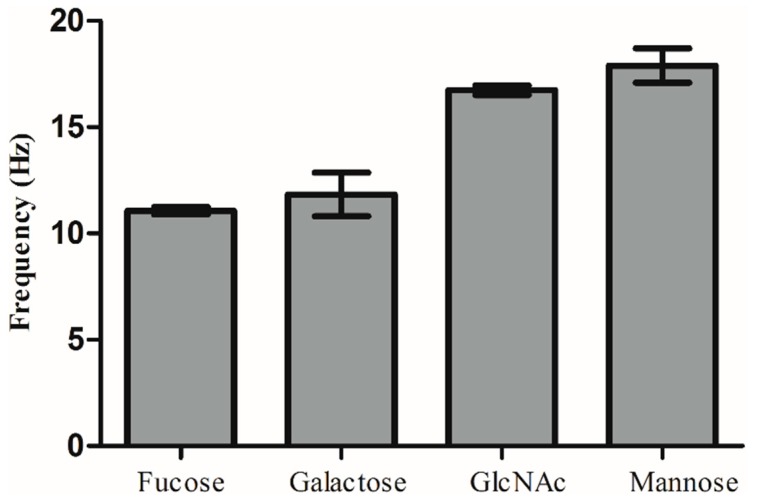
Error bar results of the four underivatized carbohydrates.

Experiments were repeated three times, and the error bar gives a general idea of how precise a measurement is. Mannose, fucose, galactose and GlcNAc were used to study specific binding with lectins under the same concentration (100 µg/mL). As shown in Figure 9, the error bar results showed that each carbohydrate and its corresponding lectin can bind specifically, illustrating the feasibility of this method.

### 3.4. Kinetic and Affinity Studies

**Figure 10:**
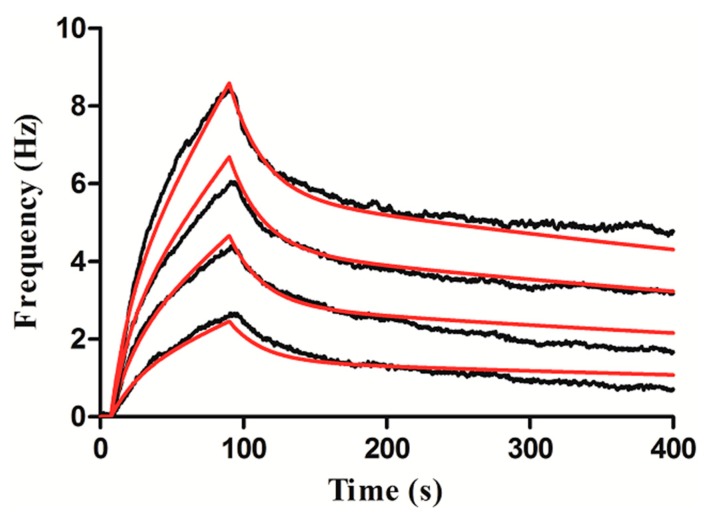
Kinetic evaluation of the interaction between fucose and UEA-I.

We used an Attana QCM Cell-A200 biosensor to perform in situ real-time measurements of the binding kinetics of the interactions between lectin and carbohydrate sensor. To study the kinetic properties of the interactions between UEA-I and fucose, a diluted solution (25, 50, 75 and 100 μg/mL) of UEA-I in the running buffer was injected to the surface. The binding was monitored in real time (the binding responses are shown in Figure 10). The overall fit was performed using a theoretical 1:1 interaction model with limitations of mass transport and employing Attana QCM Cell-A200 instrument evaluation software. Furthermore, the sensor surface was treated with 25–100 μg/mL of UEA-I, then the instrument recorded the response (black line). The Attana evaluation software generated a theoretical 1:1 fit that was covered (red line). We obtained constants of interactions between UEA-I and fucose, such as the association rate constant (K_on_ = 1.66 × 10^4^ M^−1^s^−1^), the dissociation rate constant (K_off_ = 1.27 × 10^−3^ s^−1^) and affinity constant (K_D_ = 76.5 nM).

**Figure 11:**
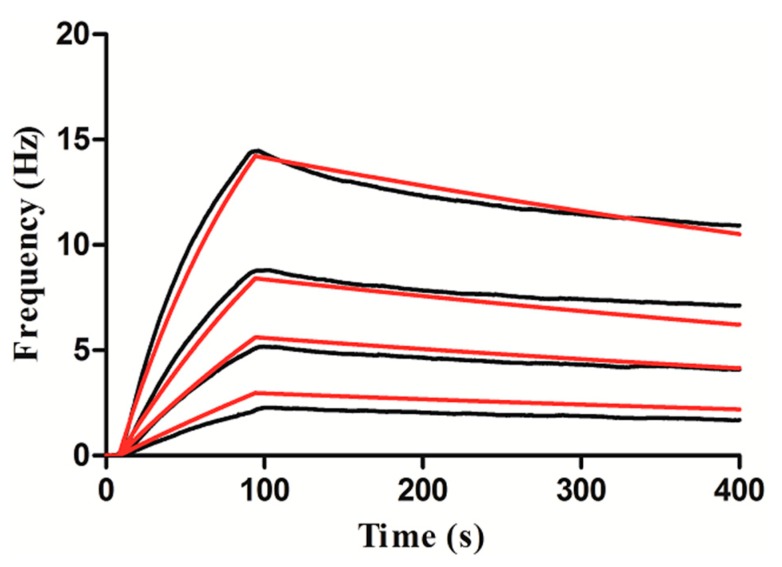
Kinetic evaluation of the interaction between mannose and Con A.

Similarly, in order to obtain the kinetic information of the interaction between Con A and mannose, a diluted Con A solution (6.25, 12.5, 20 and 40 μg/mL) was injected to the surface of the sensor. A global data fit was performed using Attana QCM Cell-A200 evaluation software, based on a theoretical 1:1 interaction model with a mass transfer limitation. As shown in Figure 11, the black line indicates the response when we injected 6.25–40 μg/mL of Con A to the surface of the sensor. It covers the theoretical 1:1 fit obtained by Attana evaluation software (red line). We obtained the constants of interactions between Con A and mannose, which are the association rate constant (K_on_ = 2.71 × 10^4^ M^−1^s^−1^), the dissociation rate constant (K_off_ = 1.05 × 10^−3^ s^−1^) and affinity constant (K_D_ = 38.8 nM).

## 4. Conclusions

In this study, a universal method for photochemical covalent fabrication of the underivatized carbohydrates to prepare the carbohydrate sensors based on PFPA modified PDA sensors for real-time detection of carbohydrate-lectin interactions by QCM biosensor was developed. The photochemical fabrication of PFPA was able to prepare carbohydrate sensors by directly inserting the underivatized carbohydrates on the sensor surfaces. The SEM-EDX energy spectrum indicated that the PFPA was successfully connected to the sensor surface. Four different underivatized carbohydrates were immobilized on the PFPA modified PDA surfaces by photochemical fabrication to prepare the carbohydrate sensors for further detecting their interactions with five different lectins (WGA, SBA, PNA, UEA-I, Con A). The experimental results show that the carbohydrate-lectin interactions are specific, which are consistent with the results obtained by other methods. A kinetic study of the interactions between mannose and Con A, fucose and UEA-I were performed. Our work provides an efficient way for the fabrication of a QCM carbohydrate sensor by underivatized carbohydrates based on PFPA modified PDA surfaces, avoiding a tedious modification of carbohydrates, which makes the QCM biosensor analysis more efficient.

## Supplementary Materials

The following are available online at https://www.mdpi.com/2073-4360/11/6/1023/s1.
