# Supplementary material for: A Universal Photochemical Method to Prepare Carbohydrate Sensors Based on Perfluorophenylazide Modified Polydopamine for Study of Carbohydrate-Lectin Interactions by QCM Biosensor
Source: Polymers (Basel). 2019 Jun 10;11(6):1023. doi: 10.3390/polym11061023 (PMC6631999; doi:10.3390/polym11061023)
Supplement: Supplementary file 1 [file polymers-11-01023-s001.pdf]

MDPI

Supporting Materials:

A Universal Photochemical Method to Prepare Carbohydrate Chips Based on Perfluorophenylazide Modified Polydopamine for Study of Carbohydrate-Lectin Interactions by QCM Biosensor

Lili Guo[1] , Shuang Chao[1], Pei Huang[1], Xiukai Lv[1], Quanquan Song[1], Chunli Wu[2, *], Yuxin Pei[1] and Zhichao Pei[1, *]

[1] Shannxi Key Laboratory of Natural Products & Chemical Biology, College of Chemistry & Pharmacy,
Northwest A & F University, Yangling 712100, China; guoll513@163.com (L.G.); chaoshuang@nwafu.edu.cn (S.C.); huangpei723@163.com (P.H.); Lvxiukai@nwafu.edu.cn (X.L.); quanquans1995@163.com (Q.S.); peiyx@nwafu.edu.cn (Y.P.)
[2] School of Pharmaceutical Sciences, Zhengzhou University, 100 Kexue Avenue, Zhengzhou 450001, China;
* Correspondence: peizc@nwafu.edu.cn; kedi2009@126.com

Synthesis and characterization of the compounds

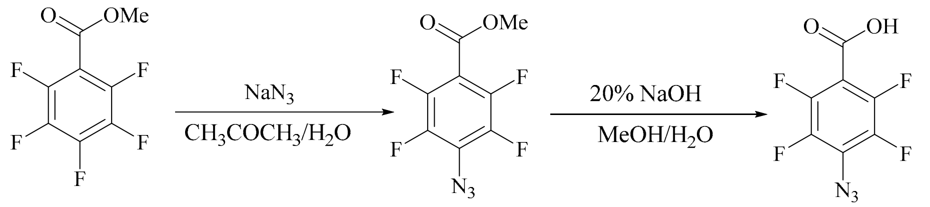

1 **2** **3**

Methyl pentafluoro-benzoate (452 mg, 2.0 mmol) was dissolved in a 8:3 (v/v) mixture of acetone and water (11 mL) of 25 mL flask. Sodium azide (140 mg, 2.15 mmol) was added to the flask and the mixture was refluxed at 65°C for 8 h in the dark, and the reaction solution is cooled to room temperature. 10 mL of water was added to the reaction solution, and extracted with diethyl ether (3 × 10 mL). The organic phase was collected, dried with over Na_2SO_4, and then filtered. The diethyl ether was evaporated under reduced pressure, obtained white crystal **2** (466.6 mg, yield 93.7%).

Compound **2** (400 mg, 1.60 mmol) was dissolved in 2 mL of methanol, add 0.5 mL of 20% aqueous sodium hydroxide solution, and react overnight at room temperature, acidified with 1 M aqueous hydrochloric acid and extracted with DCM (3 × 10 mL). The organic phase was collected and dried over anhydrous Na_2SO_4. After the end of the drying, the Na_2SO_4 was filtered off, and DCM was evaporated under reduced pressure to give Compound **3** as pale yellow solid (355.9 mg, 94.3%). ^{19}F NMR (500 MHz, $CDCl_3$, 298 K) : δ = -138.63 (m), -150.90 (m).

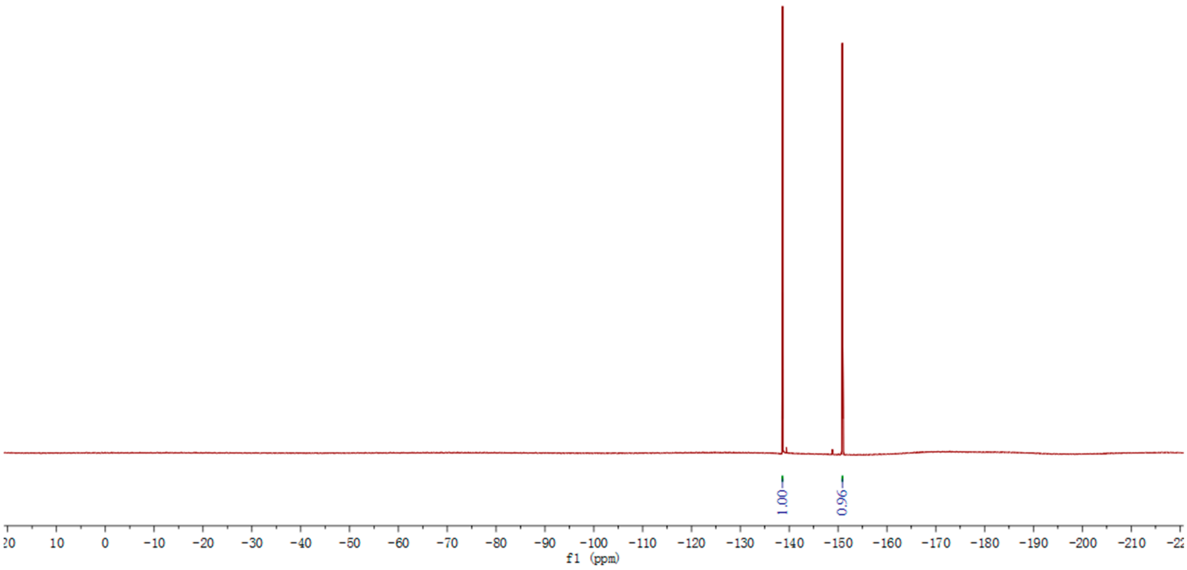

Figure S1. ^{19}F NMR (500 MHz, 298 K, $CDCl_3$) spectrum of **3**

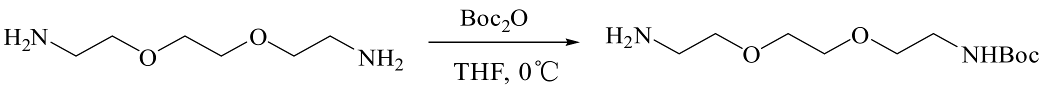

4 **5**

3.8 g (0.026 mol) of compound **4** (1,8-Diamino-3,6-dioxaoctane) and 28 mL of dry tetrahydrofuran were added to a 100 mL round bottom flask, and Boc_2O (1.7 g, 7.8 mmol) of dissolution in 16 mL of dry tetrahydrofuran was slowly added dropwise at 0°C. The reaction was overnight at room temperature, and the TLC test was completed. Removed the solvent under reduced pressure and added 35 mL of ethyl acetate, wash three times with saturated sodium chloride solution (3 × 30 mL). The organic phase was collected and dried with anhydrous Na_2SO_4. After the end of the drying, the Na_2SO_4 was filtered off, and the ethyl acetate was evaporated under reduced pressure and purified by silica gel column chromatography. The eluent was dichloromethane : methanol = 20:1 → dichloromethane : methanol = 10:1 to give compound **5** as a pale yellow oil liquid (4.4 g, 70%). ^{1}H NMR (500 MHz, $CDCl_3$, 298 K) : δ = 5.14 (s, 1H), 3.62 (s, 4H), 3.53 (m, 4H), 3.32 (q, J = 5.0 Hz, 2H), 2.89 (t, J = 5.0 Hz, 2H), 1.44 (s, 9H).

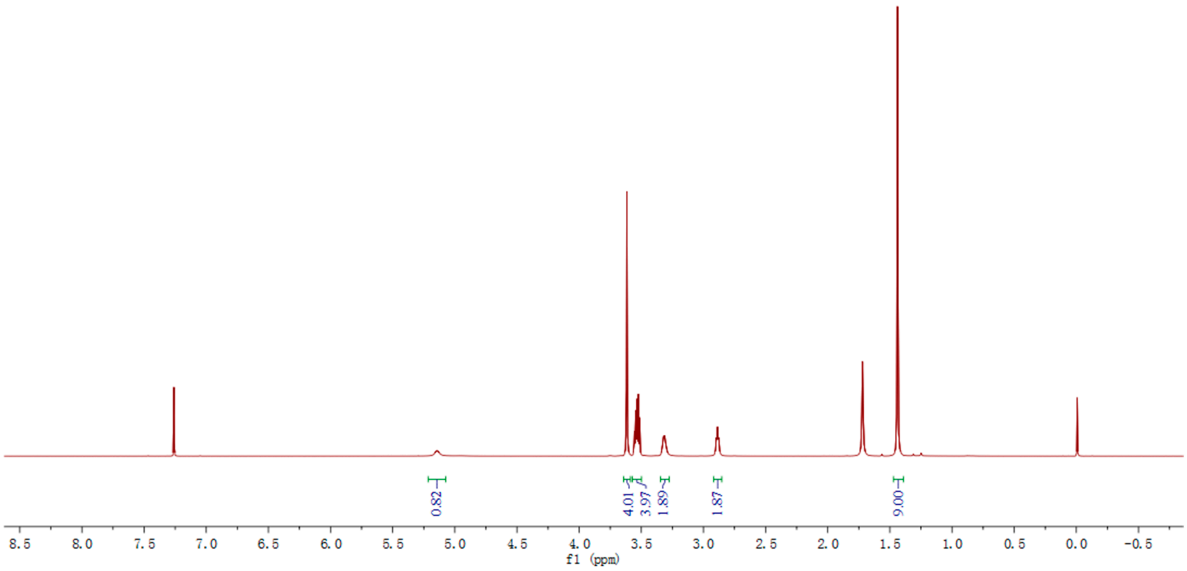

Figure S2. ^{1}H NMR (500 MHz, 298 K, $CDCl_3$) spectrum of **5**

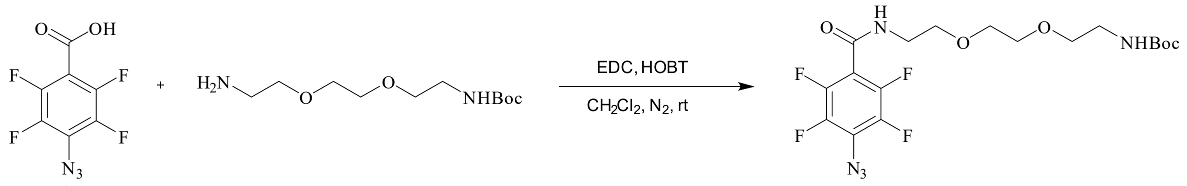

Compound **3** (141.1 mg, 0.6 mmol), EDC·HCl (343.8 mg, 1.8 mmol) and HOBT (81.1 mg, 0.6 mmol) were added to a 25 mL round bottom flask, and the system was changed to N_2 environment. 10 mL of dichloromethane was added by syringe, activated for 1 h, and compound **5** (298.0 mg, 1.2 mmol) was added, and the reaction was overnight at room temperature. After the reaction, washed three times with saturated sodium chloride solution (3 × 30 mL), the organic phase was collected and dried with anhydrous Na_2SO_4. After the end of the drying, the Na_2SO_4 was filtered off, and the dichloromethane was evaporated under reduced pressure and purified by silica gel column chromatography. The eluent was petroleum ether: ethyl acetate = 5:1 → petroleum ether: ethyl acetate = 1:1 to give compound **6** as a pale yellow oil liquid (214.5 mg, 76.8%). ^{1}H NMR (500 MHz, $CDCl_3$, 298 K) : δ = 8.68 (s, 1H), 6.85 (s, 1H), 3.72-3.57 (m, 8H), 3.57-3.45 (m, 2H), 3.30-3.16 (m, 2H), 1.47-1.27 (s, 9H). ^{13}C NMR (125 MHz, $CDCl_3$, 298 K) : δ = 157.93, 156.09, 145.14, 143.10, 141.46, 139.45, 121.85, 111.73, 79.51, 70.80, 70.58, 70.31, 69.51. MS (ESI) $C_{18}H_{23}F_4N_5O_5$ calc. for $[M + Na]^+$: 488.1533; found: 488.1529.

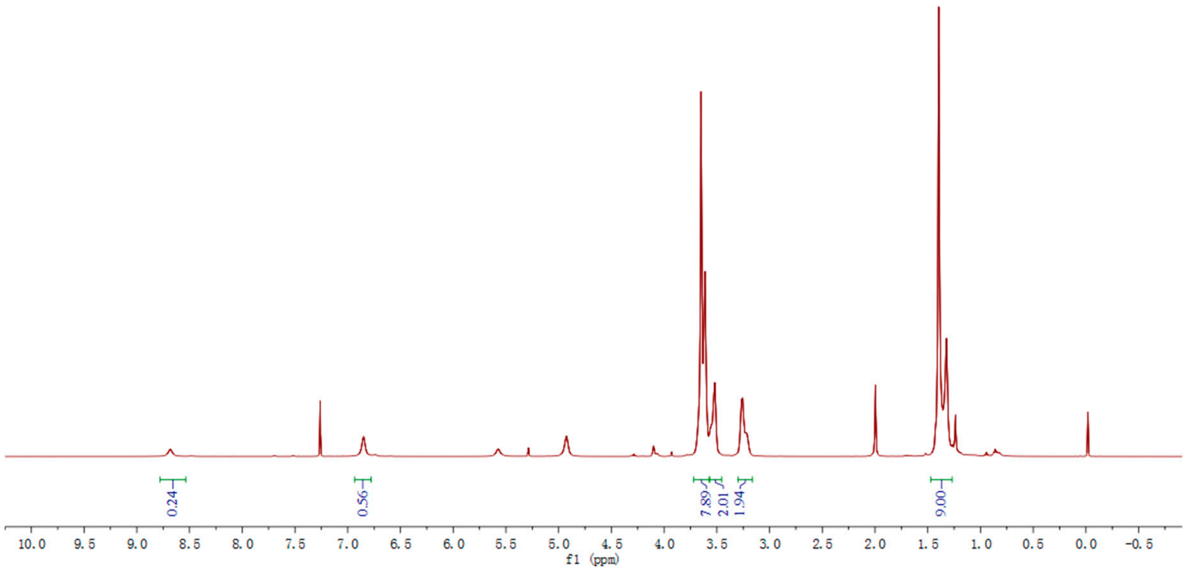

Figure S3. ^{1}H NMR (500 MHz, 298 K, $CDCl_3$) spectrum of **6**

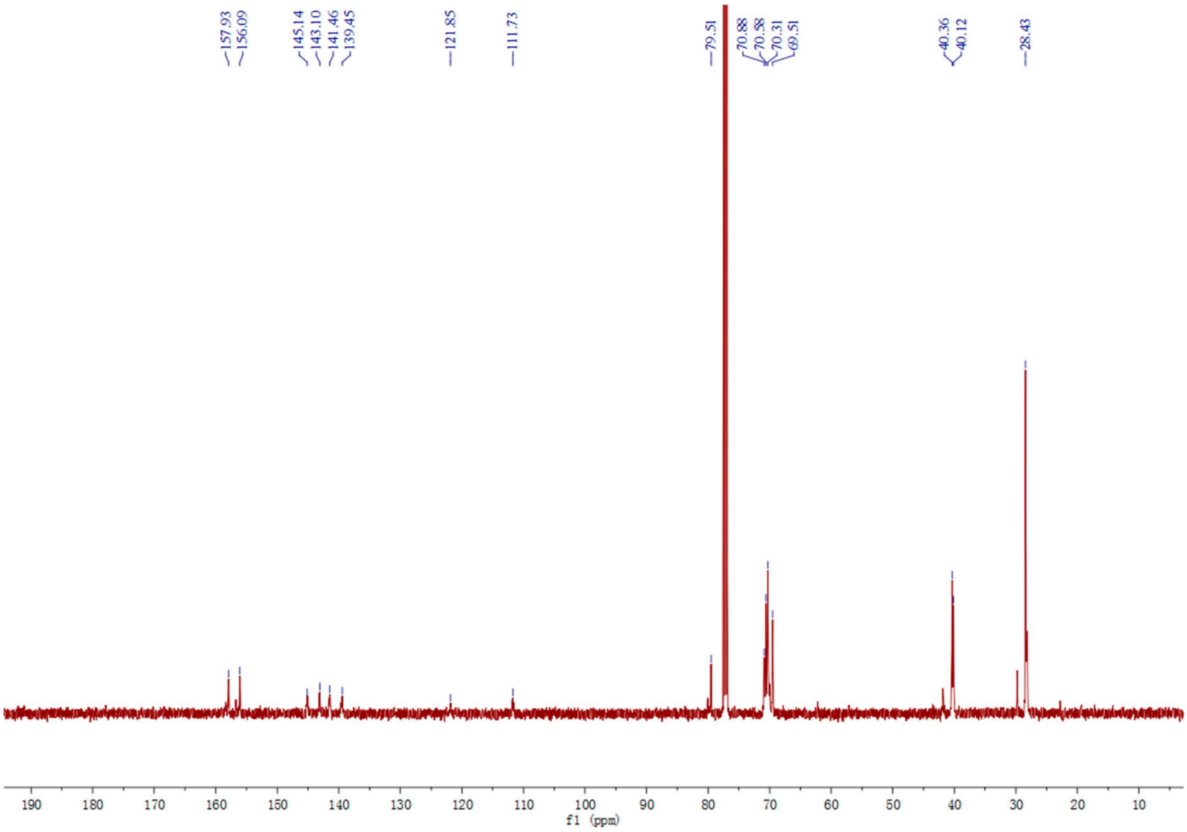

Figure S4. ^{13}C NMR (125 MHz, 298 K, $CDCl_3$) spectrum of **6**

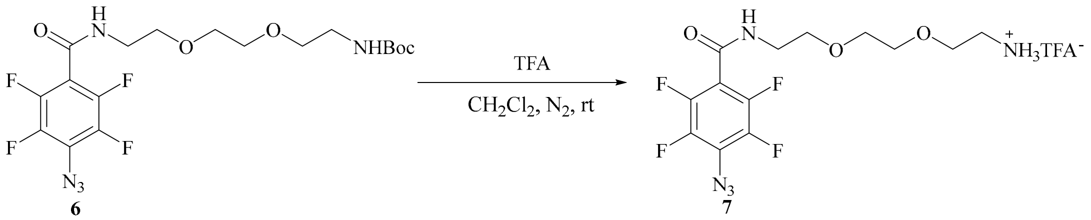

Compound **6** (176.4 mg, 0.38 mmol) was dissolved in 2 mL of dichloromethane, slowly adding trifluoroacetic acid to the reaction solution at 0°C, reacting at room temperature for 3 h. After the reaction, the dichloromethane was evaporated under reduced pressure to yield compound **7**. Compound **7** was immobilized directly on the chips without purification.

SEM-EDX spectra of different sensor surface

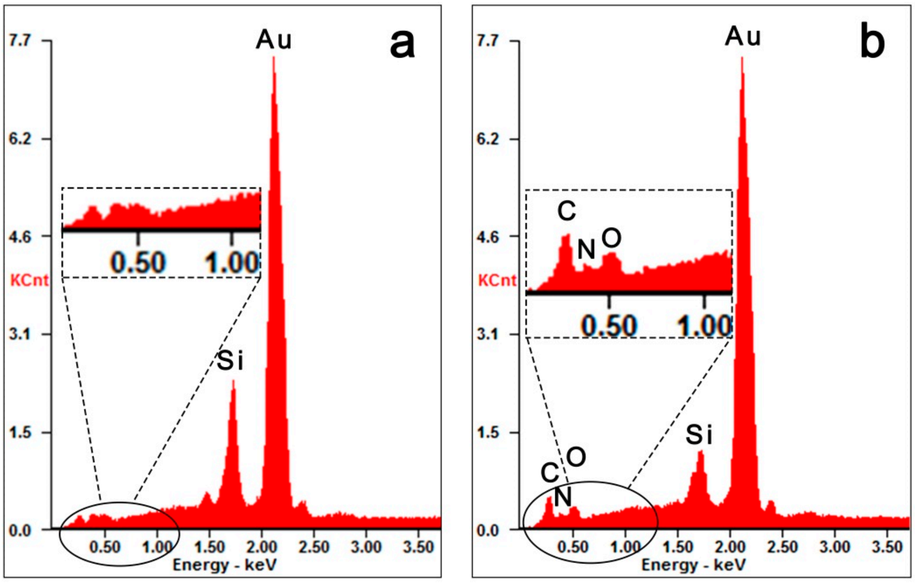

Figure S6. Region enlargement images of the peaks below 1 keV for figure 3a and 3b, (a) unmodified gold sensor surface; (b) PDA coated sensor surface.

References:

1. Lu, Y.; Song, S.; Hou, C.; Pang, S.; Pei, Z. Facile fabrication of branched-chain carbohydrate chips for studying carbohydrate-protein interactions by QCM biosensor. *Chinese Chemical Letters*. **2018**, *29*, 65-68.
